# Incidence patterns and temporal trends of childhood cancer in Germany, 1980–2019: Forty years of childhood cancer registration in Germany

**DOI:** 10.1002/ijc.70167

**Published:** 2025-09-29

**Authors:** Friederike Erdmann, Maike Wellbrock, Claudia Trübenbach, Desiree Grabow, Martin Schrappe, Peter Kaatsch, Claudia Spix, Joachim Schüz, Cécile M. Ronckers

**Affiliations:** ^1^ Research Group Aetiology and Inequalities in Childhood Cancer, Division of Childhood Cancer Epidemiology Institute of Medical Biostatistics, Epidemiology and Informatics (IMBEI), University Medical Center of the Johannes Gutenberg University Mainz Mainz Germany; ^2^ Environment and Lifestyle Epidemiology Branch, International Agency for Research on Cancer World Health Organization (IARC/WHO) Lyon France; ^3^ Division of Prevention and Evaluation Leibniz Institute for Prevention Research and Epidemiology – BIPS Bremen Germany; ^4^ Division of Childhood Cancer Epidemiology/German Childhood Cancer Registry Institute of Medical Biostatistics, Epidemiology and Informatics (IMBEI), University Medical Center of the Johannes Gutenberg University Mainz Mainz Germany; ^5^ Department of Pediatrics University Hospital Schleswig‐Holstein Kiel Germany

**Keywords:** childhood cancer, German Childhood Cancer Registry, incidence, time trends

## Abstract

Comparing incidence patterns over time and between populations stimulates aetiological research and informs health policy. With this report, we provide the first comprehensive assessment and interpretation of 40 years of childhood cancer registration data and observed incidence patterns and time trends in Germany. We identified all incident childhood cancer cases diagnosed before the age of 15 years between 1980 and 2019 from the German Childhood Cancer Registry (*N* = 65,163) and examined incidence patterns and temporal trends. Over the entire period (1980–2019), boys were more frequently diagnosed than girls, children aged <5 years had the highest age‐specific incidence rates and age‐standardised incidence rates (ASR) were highest for leukaemias and CNS tumours. Trend analyses indicated a statistically significant increase in ASRs for childhood cancer overall (from 122.8/million in 1980–1989 to 173.4/million in 2010–2019) as well as across diagnostic groups and age groups. The steepest increase (on average 4.9% per annum for all cancer diagnoses combined) occurred in the initial years of registration (1980–1987), mostly driven by the sharp increase in the reporting of CNS tumours diagnoses, soft tissue sarcoma and germ cell tumours. Since the 1990s, temporal patterns were more heterogeneous across diagnostic groups; yet overall, less pronounced than during the build‐up phase of the registry. Various factors have influenced the observed incidence patterns in Germany over the 40‐year registration period. The steep increase in ASRs during the early years is primarily attributable to improvements in case reporting and registration. Explanations for the more recent temporal patterns remain speculative.

## Graphical abstract

What's new?

Childhood cancer ranks among the leading causes of disease‐related deaths in children in high‐income countries. Established risk factors, however, account for only a small proportion of incident childhood cancers. In this report, the authors present the first long‐term assessment of temporal trends in childhood cancer incidence rates in Germany, covering a 40‐year period and a study population representing more than 450 million person‐years at risk. This makes it one of the largest reports worldwide, offering valuable insights into reporting practices, incidence trends, and factors that may have influenced these patterns at different points over the four decades.

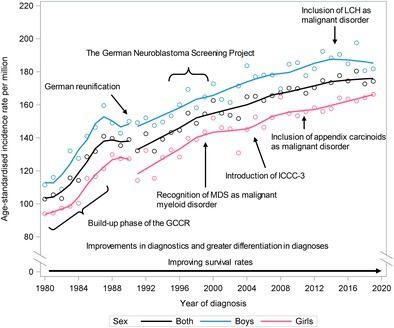

**Abbreviations**
AAPC: average annual percentage change
APC: annual percentage changes
ASR: age‐standardized incidence rate
CI: confidence interval
CNS: central nervous system
GCCR: German Childhood Cancer Registry
ICCC‐3: International Classification of Childhood Cancer (3rd edition)
LCH: Langerhans cell histiocytosis
LL: lymphoid leukaemia
MDS: myelodysplastic syndrome

## 1 INTRODUCTION

Although childhood cancer is relatively rare compared to cancer in adults, it is the leading cause of disease‐related deaths among children aged 1–14 years in high‐income countries (HIC).^1^ Childhood cancer is a heterogeneous group of neoplasia, with leukaemias and tumours of the central nervous system (CNS) representing the most frequent diagnoses in 0–14 year olds in Europe and other HIC.^2^, ^3^, ^4^ The aetiology of most childhood cancers remains largely unknown and preventive measures are lacking.^5^, ^6^ A growing body of research has assessed a broad range of potential risk factors including pregnancy‐related factors,^7^, ^8^, ^9^ exposure to infections,^8^, ^10^ environmental pollutants,^11^, ^12^, ^13^, ^14^ occupational exposures,^15^, ^16^ and parental lifestyle^14^, ^17^, ^18^, ^19^ with, however, largely inconsistent and yet inconclusive results. To date, the established risk factors account for only a small fraction (<10%) of all incident childhood cancer cases and include certain chromosomal and genetic conditions, birth defects in general, exposure to high‐dose ionising radiation, advanced maternal age at the child's birth as well as high and low birth weight.^6^, ^10^, ^14^, ^20^, ^21^, ^22^ Although treatment of and survival from childhood cancer has considerably improved over the last decades,^23^, ^24^ the cancer diagnosis and its treatment are a distressing experience for the entire family, with potentially far‐reaching and lifelong adverse somatic late effects as well as psychosocial and socioeconomic consequences for the former patients and their families.^23^ Moreover, some neoplastic diseases remain fatal in more than half of initially affected children, or when aggressive, fast‐growing recurrences occur. Hence, gaining a better understanding of the causes of childhood cancer to implement primary preventive measures remains the ultimate goal.

Examining epidemiological patterns, including differences in incidence between age groups, between regions and countries, and over time, may provide useful indications for aetiological research and is of high importance for planning and organisation of healthcare. The German Childhood Cancer Registry (GCCR) is one of the few long‐standing national childhood cancer registries in the world. It was first established in the Federal Republic of Germany (“West Germany”) in 1980 and was expanded following the German reunification (in 1991) to cover the entire territory of unified Germany, including the former German Democratic Republic.

Here we provide the first comprehensive assessment and interpretation of reported incidence data and observed temporal trends in childhood cancer in Germany, based on 40 years of data from the German Childhood Cancer Registry. Although the incidence of most cancer types was likely affected by underreporting during the registry's build‐up phase, the longitudinal data offer high granularity, which we aimed to take advantage of. In addition, we critically discuss factors that may have influenced the observed incidence patterns at different points over the 40‐year registration period.

## 2 MATERIALS AND METHODS

### 2.1 Case definition and registration procedure at the German Childhood Cancer Registry

We identified all incident cancer cases (including non‐malignant intracranial and intraspinal tumours) diagnosed in children aged 0–14 years with German residency between 1 January 1980 and 31 December 2019 from the GCCR. We deliberately defined the end of the study period prior to the onset of the COVID‐19 pandemic to avoid any influence of the pandemic on incidence estimates; such effects have been described elsewhere.^25^, ^26^

The GCCR was established in 1980 at the institute now known as the Institute of Medical Biostatistics, Epidemiology and Informatics at the University Medical Center Mainz, part of Johannes Gutenberg University. It is the national childhood cancer registry of Germany, monitoring incident diagnoses of cancers in children aged 0–14 years (since 2009 expanded to 0–17 years). The registration process of the GCCR is based on voluntary reporting by all paediatric haematology‐oncology units in Germany; the number of units varied somewhat over time, for example, from 63 to 59 units in 2015–2020 (with a decreasing tendency) and written informed consent of the respective parents and patients. In case no written consent is given, incident cancer diagnoses are registered without any personal identifiable information. During the initial build‐up phase until about 1987, the GCCR quickly established a close and well‐functioning network with treating hospitals and the Society of Paediatric Oncology and Haematology (GPOH), the association of paediatric oncology professionals in Germany, which guarantees the coverage of virtually all paediatric cancer cases that are diagnosed and treated in a paediatric oncology unit. Some patients, primarily adolescents or those not requiring chemotherapy, are treated in adult oncological or neuro‐oncological facilities, which do not always report reliably to the GCCR. Excellent population‐based coverage for almost all cancer types is achieved since about 1987, with completeness of registration being estimated to exceed 95% (for children aged 0–14 years, all cancer types combined), accomplished through the close collaboration with the GPOH, treating hospitals and clinical trials. The high level of completeness was ensured by annual comparisons of newly diagnosed patients with virtually all treating hospitals, particularly during the first two decades of the registry.^27^

Childhood cancer diagnoses were defined according to the International Classification of Childhood Cancer‐third edition (ICCC‐3).^28^ Because symptoms and prognosis of intracranial and intraspinal tumours of non‐malignant or uncertain behaviour often resemble those of malignant tumours, they are included in the ICCC and, consequently, in the GCCR. Cancer diagnoses reported to the GCCR were initially classified according to the first edition of the International Classification of Childhood Cancer (ICCC) by Birch and Marsden.^29^ Following the release of each new edition of the International Classification of Diseases for Oncology (ICD‐O), all diagnoses were retrospectively recoded and reclassified according to the corresponding ICCC edition. Since 2005, classifications and reclassifications have been based on the third edition, ICCC‐3.^28^ Updates to the childhood cancer classification system led to the inclusion of a few additional neoplasms that had not previously been classified as childhood cancers (with the introduction of ICD‐O‐3 and, subsequently, ICD‐O‐3.1). Given their small numbers, these additional diagnoses had only a negligible impact on overall incidence rates. The most notable additions are myelodysplastic syndrome (MDS), Langerhans cell histiocytosis (LCH) and appendix carcinoids.^28^

Annual population estimates are regularly obtained from The Federal Statistical Office of Germany. Since the reunification in 1991, the population of children under 15 years of age in Germany has ranged between 10.6 and 13.3 million, peaking in 1994 at 13.298 million and reaching its lowest point in 2013 at 10.628 million. During the 1980s, the childhood population in West Germany was approximately 9.7 million. Over the entire observation period from 1980 to 2019, this corresponds to a total of more than 450 million person‐years at risk.

### 2.2 Statistical analyses

To evaluate indices of quality, we calculated the proportions of microscopically verified diagnoses that were confirmed from histology of a primary tumour, haematological examination of peripheral blood/bone marrow, or histology of metastasis. We calculated the proportions of subsequent primary neoplasms (SPN) (any new primary tumours diagnosed in a child aged 0–14 years with a previous cancer diagnosis), average numbers of yearly cases, age‐specific and age‐standardised incidence rates (ASR), male‐to‐female ratios, the cumulative incidence (defined as the sum of age‐specific incidence rates over each year of age from 0 to 14 years) as well as the risk of a cancer diagnosis before the age of 15 years, by ICCC‐3 diagnostic groups and by diagnostic period to describe the incidence patterns of childhood cancer in Germany. We calculated ASRs by applying the weights of the Segi 1960 World Standard Population (age groups <1, 1–4, 5–9 and 10–14 years)^30^ and expressed ASRs per million children. Corresponding 95% confidence intervals (CI) were calculated assuming Poisson distribution. To reflect the change in the population covered by the GCCR since 1991 (after the German reunification), diagnostic periods were grouped as follows: 1980–1990, 1991–1999, 2000–2009 and 2010–2019.

To portray incidence trends graphically, we plotted the ASRs for all childhood cancers combined and for selected diagnostic groups over time, applying a locally estimated scatterplot smoothing (LOESS) with a smoothing parameter of 0.4 and linear interpolation to the ASRs by calendar year. For time‐trend analyses, we used Joinpoint regression analysis.^31^ This method allowed us to calculate the (average) annual percentage changes ((A)APC) in incidence rates and to evaluate whether the magnitude or direction of trends changed during the study period. The Joinpoint model applies Monte Carlo permutation tests to detect points in time marking significant changes in magnitude or direction of temporal trends (the so‐called joinpoints); the year in which such a significant change is detected is termed “joinpoint.” An APC is considered as the percentage change per year between two time segments identified in Joinpoint regression analysis; the AAPCs reflect the average change over a longer period (e.g., start to end of the study period). To reduce the likelihood of reporting spurious changes in trends, we allowed up to four joinpoints during the entire study period.

As a sensitivity analysis, we reran the Joinpoint regression excluding cases that had not previously been classified as childhood cancers but are considered malignant in the updated classification system in order to assess the impact of these additional neoplasms on the observed time trends.

Analyses were performed using SAS® Software 9.4^32^ and Joinpoint Regression Program, version 4.9.0.0, National Cancer Institute.^31^

## 3 RESULTS

In total, 65,163 incident cases of cancer in children aged 0–14 years were reported to the GCCR over the 40‐year period from 1980 to 2019 (Table 1), representing more than 450 million person‐years at risk. The average number of annual reported cases increased steadily over time from 1126 in 1980–1990 to 1834 in 2010–2019. The proportion of microscopically verified cases stabilised at around 95% since the 1990s, when clinical trials had been initiated for the majority of diagnoses. Lower verification rates were observed for CNS tumours and retinoblastomas, for which clinical diagnoses are accepted in cancer registration, while the highest rate was seen for lymphoid leukaemia, with 100% microscopic confirmation. SPNs made up a small fraction of all incident cases (ranging from 0.4% to 1.4% across diagnostic groups).

**TABLE 1:** Counts of incident cancer cases in children aged 0–14 years in Germany in 1980–2019, proportions of microscopically verified diagnoses and proportion of subsequent primary neoplasms, by diagnostic period.

	1980–1990				1991–1999			
	Cases, *N*	Average annual cases (%)	Microscopically verified^a^ (%)	Subsequent primary neoplasms^b^ (%)	Cases, *N*	Average annual cases (%)	Microscopically verified^a^ (%)	Subsequent primary neoplasms (%)
All cancer diagnoses	12,382	1125.6 (100.0)	85.7	0.4	16,151	1794.6 (100.0)	95.4	1.0
ICCC‐3 diagnostic group^c^								
Leukaemias	4464	405.8 (36.1)	99.96	0.4	5515	612.8 (34.1)	99.9	1.0
Lymphoid leukaemia	3610	328.2 (29.2)	100.0	0.1	4460	495.6 (27.6)	100.0	0.2
Lymphomas	1469	133.5 (11.9)	96.7	0.4	2023	224.8 (12.5)	99.6	1.1
CNS tumours	2250	204.5 (18.2)	50.4	0.5	3323	369.2 (20.6)	84.4	1.4
Malignant	1529	139.0 (12.3)	53.8	0.5	2112	234.7 (13.1)	84.6	1.6
Non‐malignant^d^	721	65.5 (5.8)	43.1	0.3	1211	134.6 (7.5)	83.9	1.0
Neuroblastoma	947	86.1 (7.6)	95.0	0.0	1324	147.1 (8.2)	99.5	0.2
Retinoblastoma	357	32.5 (2.9)	92.7	0.3	366	40.7 (2.3)	83.1	0.3
Renal tumours	776	70.5 (6.3)	89.4	0.0	991	110.1 (6.1)	96.8	0.2
Hepatic tumours	134	12.2 (1.1)	43.3	0.0	130	14.4 (0.8)	96.9	0.0
Bone tumours	674	61.3 (5.4)	87.1	0.3	726	80.7 (4.5)	98.2	1.3
Soft tissue sarcomas	823	74.8 (6.6)	83.1	0.9	1002	111.3 (6.2)	97.2	0.7
Germ cell tumours	368	33.5 (2.8)	83.2	0.0	557	61.9 (3.4)	97.5	0.5
Epithelial tumours and melanomas	109	9.9 (0.9)	22.9	2.9	177	19.7 (1.1)	75.7	8.2
Other malignant neoplasms	11	1.0 (0.1)	81.8	0.0	17	1.9 (0.1)	88.2	0.0
Age at diagnosis (years)								
<1	1225	111.4 (9.9)	84.8	0.0	1614	179.3 (10.0)	94.7	0.1
1–4	4645	422.3 (37.5)	88.9	0.2	5850	650.0 (36.2)	96.4	0.3
5–9	3179	289.0 (25.7)	84.9	0.5	4565	507.2 (28.3)	94.7	1.5
10–14	3333	303.0 (26.9)	82.4	0.7	4122	458.0 (25.5)	95.2	1.8

	2000–2009				2010–2019			
	Cases, *N*	Average annual cases (%)	Microscopically verified^a^ (%)	Subsequent primary neoplasms (%)	Cases, *N*	Average annual cases (%)	Microscopically verified^a^ (%)	Subsequent primary neoplasms (%)
All cancer diagnoses	18,289	1828.9 (100.0)	94.7	1.4	18,341	1834.1 (100.0)	94.3	1.1
ICCC‐3 diagnostic group^b^								
Leukaemias	6140	614.0 (33.6)	99.8	1.3	5749	574.9 (31.3)	99.7	1.1
Lymphoid leukaemia	4805	480.5 (26.3)	99.9	0.2	4430	443.0 (24.2)	99.9	0.4
Lymphomas	2062	206.2 (11.3)	99.5	1.1	2193	219.3 (12.0)	99.8	1.0
CNS tumours	4333	433.3 (23.7)	84.3	1.2	4645	464.5 (25.3)	81.1	0.9
Malignant	2541	254.1 (13.9)	79.1	1.5	2680	268.0 (14.6)	72.2	1.1
Non‐malignant^c^	1992	179.2 (9.8)	91.6	0.7	1965	196.5 (10.7)	93.4	0.5
Neuroblastoma	1297	129.7 (7.1)	99.5	0.3	1187	118.7 (6.5)	99.3	0.4
Retinoblastoma	411	41.1 (2.2)	45.3	0.2	449	44.9 (2.4)	78.0	0.0
Renal tumours	1020	102.0 (5.8)	99.4	0.8	942	94.2 (5.1)	99.5	0.4
Hepatic tumours	221	22.1 (1.2)	93.2	0.9	274	27.4 (1.5)	92.3	0.4
Bone tumours	811	81.1 (4.4)	99.9	2.7	793	79.3 (4.3)	99.5	3.2
Soft tissue sarcomas	1102	110.2 (6.0)	99.8	1.8	976	97.6 (5.3)	99.6	0.9
Germ cell tumours	548	54.8 (3.0)	99.5	0.2	614	61.4 (3.3)	98.4	0.0
Epithelial tumours and melanomas	323	32.3 (1.8)	98.5	10.5	486	48.6 (2.6)	98.6	6.2
Other malignant neoplasms	21	2.1 (0.1)	90.5	14.3	33	3.3 (0.2)	100.0	0.0
Age at diagnosis (years)								
<1	1869	186.9 (10.2)	90.6	0.1	1953	195.3 (10.6)	94.1	0.2
1–4	6230	623.0 (34.1)	94.5	0.6	6354	635.4 (34.6)	94.1	0.4
5–9	4897	489.7 (26.8)	94.5	1.5	4694	469.4 (25.7)	93.5	1.4
10–14	5293	529.3 (28.9)	96.6	2.6	5340	534.0 (29.1)	95.2	2.0

^a^ Microscopically verified cases.

^b^ Subsequent primary neoplasms were defined as any new primary tumours diagnosed in a child (0–14 years of age) with a previous cancer diagnosis, regardless of the time between the two diagnoses.

^c^ Diagnostic groups defined according to the International Classification of Childhood Cancer‐3rd edition (ICCC‐3).

^d^ Intracranial and intraspinal tumours with non‐malignant behaviour (defined according to ICD‐O‐3).

The observed annual ASR for all cancer types combined increased gradually from 122.8 per million children in 1980–1989 to 173.4 per million in 2010–2019 (Table 2); correspondingly, the cumulative incidence rose from 1766.4 to 2520.0 per million in 2010–2019. Throughout the study period, ASRs were highest for leukaemias, followed by CNS tumours and lymphomas and boys were somewhat more frequently diagnosed than girls (sex ratio of 1.23 in 2000–2019) (Table 2). In 2010–2019, the ASRs for leukaemias, CNS tumours and lymphomas were 55.5, 43.2 and 19.0 per million children, respectively. This corresponds to an estimated risk of 1 in 1259 children being diagnosed with leukaemia before the age of 15, 1 in 1566 with a CNS tumour and 1 in 3359 with lymphoma. ASRs for solid tumours outside the CNS ranged from 12.7 per million for neuroblastoma to 2.9 per million for hepatic tumours (Table 2), with correspondingly lower risks of diagnosis. The sex ratio varied considerably by cancer type and was largest for lymphomas (sex ratio = 2.00).

**TABLE 2:** Incidence of childhood cancer (diagnosed at ages 0–14 years) in Germany in 1980–2019, by diagnostic period and diagnostic group.

	ASR per million^a^ (95% CI)	Cumulative incidence per million (95% CI)	Risk of childhood cancer^b^	Male/female^c^
1980–1990				
All cancer diagnoses	122.8 (120.6–125.0)	1766.4 (1735.3–1797.7)	566	1.29
ICCC‐3 diagnostic group^d^				
Leukaemias	45.1 (43.8–46.5)	644.7 (625.9–663.8)	1551	1.27
Lymphoid leukaemia	36.9 (35.7–38.1)	524.9 (507.9–542.2)	1905	1.29
Lymphomas	12.8 (12.2–13.5)	200.7 (190.5–211.1)	4982	2.17
CNS tumours	21.9 (20.9–22.8)	322.0 (308.8–335.4)	3106	1.27
Malignant	15.1 (14.3–15.8)	219.9 (209.0–231.1)	4547	1.36
Non‐malignant	6.8 (6.3–7.3)	102.1 (94.7–109.7)	9799	1.10
Neuroblastoma	10.7 (10.0–11.4)	139.5 (130.8–148.6)	7167	1.20
Retinoblastoma	4.1 (3.7–4.5)	52.7 (47.4–58.3)	18,984	1.08
Renal tumours	8.6 (8.0–9.2)	115.2 (107.3–123.5)	8677	0.98
Hepatic tumours	1.4 (1.2–1.7)	19.2 (16.1–22.6)	51,961	1.39
Bone tumours	5.5 (5.1–5.9)	88.7 (82.1–95.5)	11,279	1.07
Soft tissue sarcomas	8.0 (7.5–8.6)	116.2 (108.4–124.3)	8604	1.25
Germ cell tumours	3.7 (3.3–4.1)	51.5 (46.4–56.9)	19,416	0.95
Epithelial tumours and melanomas	0.9 (0.7–1.1)	14.3 (11.8–17.2)	69,793	1.10
Other malignant neoplasms	0.1 (0.1–0.2)	1.6 (0.8–2.6)	641,207	1.20
1991–1999				
All cancer diagnoses	143.4 (141.1–145.6)	2072.5 (2040.6–2104.6)	483	1.27
ICCC‐3 diagnostic group				
Leukaemias	49.5 (48.2–50.8)	706.8 (688.3–725.6)	1415	1.32
Lymphoid leukaemia	40.3 (39.1–41.5)	571.3 (554.7–588.2)	1750	1.37
Lymphomas	16.0 (15.3–16.7)	253.1 (242.2–264.3)	3951	1.92
CNS tumours	28.6 (27.7–29.6)	422.6 (408.4–437.1)	2366	1.22
Malignant	18.4 (17.6–19.2)	269.1 (257.7–280.7)	3716	1.29
Non‐malignant	10.3 (9.7–10.9)	153.5 (145.0–162.3)	6514	1.11
Neuroblastoma	13.6 (12.8–14.3)	177.7 (168.2–187.4)	5628	1.18
Retinoblastoma	3.8 (3.4–4.2)	49.4 (44.5–54.6)	20,234	0.92
Renal tumours	9.7 (9.1–10.3)	130.1 (122.1–138.3)	7687	0.95
Hepatic tumours	1.3 (1.1–1.5)	17.4 (14.5–20.5)	57,551	2.25
Bone tumours	5.5 (5.1–5.9)	90.4 (83.9–97.1)	11,066	1.17
Soft tissue sarcomas	8.8 (8.2–9.3)	128.2 (120.3–136.2)	7703	1.24
Germ cell tumours	5.0 (4.6–5.5)	72.5 (66.6–78.6)	13,798	0.74
Epithelial tumours and melanomas	1.4 (1.2–1.6)	22.1 (19.0–25.5)	45,179	0.90
Other malignant neoplasms	0.2 (0.1–0.2)	2.2 (1.3–3.4)	451,110	0.89
2000–2009				
All cancer diagnoses	161.6 (159.2–164.0)	2340.3 (2306.4–2374.5)	427	1.23
ICCC‐3 diagnostic group^b^				
Leukaemias	55.4 (54.0–56.8)	791.2 (771.5–811.1)	1264	1.21
Lymphoid leukaemia	43.6 (42.4–44.9)	620.7 (603.3–638.4)	1611	1.23
Lymphomas	15.7 (15.0–16.4)	250.0 (239.3–260.9)	3359	1.97
CNS tumours	37.4 (36.2–38.5)	551.1 (534.7–567.6)	1566	1.23
Malignant	22.2 (21.3–23.1)	324.7 (312.1–337.4)	3080	1.33
Non‐malignant	15.2 (14.5–15.9)	226.4 (216.0–237.0)	4417	1.11
Neuroblastoma	13.6 (12.9–14.4)	177.9 (168.3–187.7)	6077	1.20
Retinoblastoma	4.4 (4.0–4.8)	56.8 (51.5–62.5)	16,069	1.22
Renal tumours	10.1 (9.5–10.7)	136.5 (128.2–145.0)	7326	0.91
Hepatic tumours	2.2 (1.9–2.5)	29.5 (25.8–33.6)	33,848	1.63
Bone tumours	5.9 (5.5–6.4)	96.6 (90.1–103.4)	10,349	1.09
Soft tissue sarcomas	9.6 (9.0–10.1)	140.0 (131.8–148.4)	7145	1.20
Germ cell tumours	4.7 (4.3–5.2)	69.5 (63.8–75.4)	14,393	0.79
Epithelial tumours and melanomas	2.4 (2.1–2.7)	38.6 (34.5–43.0)	25,891	0.78
Other malignant neoplasms	0.2 (0.1–0.3)	2.6 (1.6–3.9)	381,777	0.75
2010–2019				
All cancer diagnoses	173.4 (170.9–176.0)	2520.0 (2483.7–2556.7)	397	1.23
ICCC‐3 diagnostic group				
Leukaemias	55.4 (54.0–56.9)	794.5 (774.1–815.2)	1259	1.24
Lymphoid leukaemia	43.0 (41.8–44.3)	613.9 (595.9–632.1)	1629	1.27
Lymphomas	19.0 (18.3–19.9)	297.7 (285.4–310.3)	3359	2.00
CNS tumours	43.1 (41.9–44.4)	638.7 (620.4–657.2)	1566	1.17
Malignant	25.3 (24.3–26.3)	369.5 (355.7–383.7)	2706	1.20
Non‐malignant	17.8 (17.0–18.6)	269.1 (257.3–281.2)	3716	1.13
Neuroblastoma	12.7 (12.0–13.4)	164.6 (155.3–174.1)	6077	1.31
Retinoblastoma	4.9 (4.4–5.3)	62.2 (56.6–68.1)	16,069	1.11
Renal tumours	9.7 (9.1–10.3)	131.1 (122.8–139.6)	7630	0.89
Hepatic tumours	2.9 (2.5–3.2)	37.8 (33.5–42.4)	26,437	1.28
Bone tumours	6.5 (6.1–7.0)	106.5 (99.2–114.0)	9394	1.15
Soft tissue sarcomas	9.2 (8.6–9.8)	134.1 (125.8–142.6)	7459	1.25
Germ cell tumours	5.7 (5.2–6.1)	83.3 (76.8–90.0)	12,006	0.80
Epithelial tumours and melanomas	4.0 (3.6–4.3)	65.0 (59.4–70.9)	15,377	0.75
Other malignant neoplasms	0.3 (0.2–0.5)	4.6 (3.1–6.2)	219,296	0.94

^a^ ASR: age‐standardized incidence rate (standardized according to the Segi World Standard Population) per 1,000,000 children aged 0–14 years.

^b^ Risk of a cancer diagnosis before the age of 15 years (calculation: 1 divided by the cumulative incidence per million × 1 million).

^c^ Absolute number of male cases/absolute number of female cases.

^d^ Diagnostic groups defined according to the International Classification of Childhood Cancer‐3rd edition (ICCC‐3).

Based on data from the most recent diagnostic period (2010–2019), the highest age‐specific incidence rate for childhood cancer overall was observed in infants (270 per million), followed by children aged 1–4 years (222 per million), while the lowest rate was seen in 5–9 year olds (132 per million) (Table 3). The age distribution varied substantially between diagnostic groups; leukaemias had the highest incidence rate in 1–4 year olds, lymphomas occurred more often in older children, while neuroblastoma, retinoblastoma, renal tumours, hepatic tumours and germ cell tumours occurred mostly in infants and CNS tumours' incidence rates did not vary that strongly by age (Table 3). Consequently, the distribution of diagnostic groups varied between age groups.

**TABLE 3:** Age‐specific incidence rates of cancer in children aged 0–14 years in Germany in 2010–2019, by diagnostic group.

	Age‐specific incidence rate (95% CI)			
	<1 year	1–4 years	5–9 years	10–14 years
All cancer diagnoses	269.9 (258.0–282.1)	221.7 (216.3–227.2)	131.6 (127.8–135.4)	141.4 (137.7–145.3)
ICCC‐3 diagnostic group^a^				
Leukaemias	43.8 (39.1–48.9)	90.7 (87.2–94.2)	45.2 (43.0–47.4)	32.4 (30.6–34.2)
Lymphoid leukaemia	16.9 (14.0–20.1)	76.9 (73.7–80.1)	36.6 (34.6–38.6)	21.2 (19.7–22.7)
Lymphomas	8.7 (6.7–11.1)	13.0 (11.7–14.4)	17.9 (16.5–19.4)	29.6 (27.9–31.4)
CNS tumours	42.1 (37.6–47.2)	50.0 (47.5–52.7)	41.8 (39.7–44.0)	37.5 (35.5–39.5)
Malignant	28.2 (24.5–32.3)	32.6 (30.5–34.7)	24.0 (22.4–25.7)	18.2 (16.8–19.6)
Non‐malignant	14.0 (11.4–17.0)	17.5 (16.0–19.1)	17.8 (16.4–19.2)	19.3 (17.9–20.8)
Neuroblastoma	73.5 (67.4–80.0)	18.6 (17.0–20.2)	2.5 (2.0–3.1)	0.9 (0.6–1.3)
Retinoblastoma	28.7 (25.0–32.9)	8.0 (7.0–9.1)	0.3 (0.1–0.5)	0.1 (0.0–0.2)
Renal tumours	19.9 (16.8–23.4)	18.8 (17.3–20.5)	5.7 (4.9–6.5)	1.5 (1.1–1.9)
Hepatic tumours	11.1 (8.8–13.8)	5.2 (4.4–6.1)	0.5 (0.3–0.8)	0.7 (0.4–1.0)
Bone tumours	0.6 (0.2–1.4)	1.9 (1.4–2.5)	5.3 (4.6–6.1)	14.4 (13.2–15.7)
Soft tissue sarcomas	17.1 (14.3–20.4)	10.3 (9.2–11.6)	7.4 (6.5–8.3)	7.7 (6.9–8.7)
Germ cell tumours	22.7 (19.3–26.4)	3.2 (2.6–4.0)	2.4 (1.9–2.9)	7.2 (6.4–8.1)
Epithelial neoplasms and melanomas	1.0 (0.4–2.0)	1.1 (0.8–1.6)	2.6 (2.1–3.2)	9.3 (8.4–10.4)
Other malignant neoplasms	0.7 (0.2–1.6)	0.8 (0.5–1.2)	0.03 (0.0–0.2)	0.1 (0.0–0.3)

^a^ Diagnostic groups defined according to the International Classification of Childhood Cancer‐3rd edition (ICCC‐3).

**FIGURE 1:**
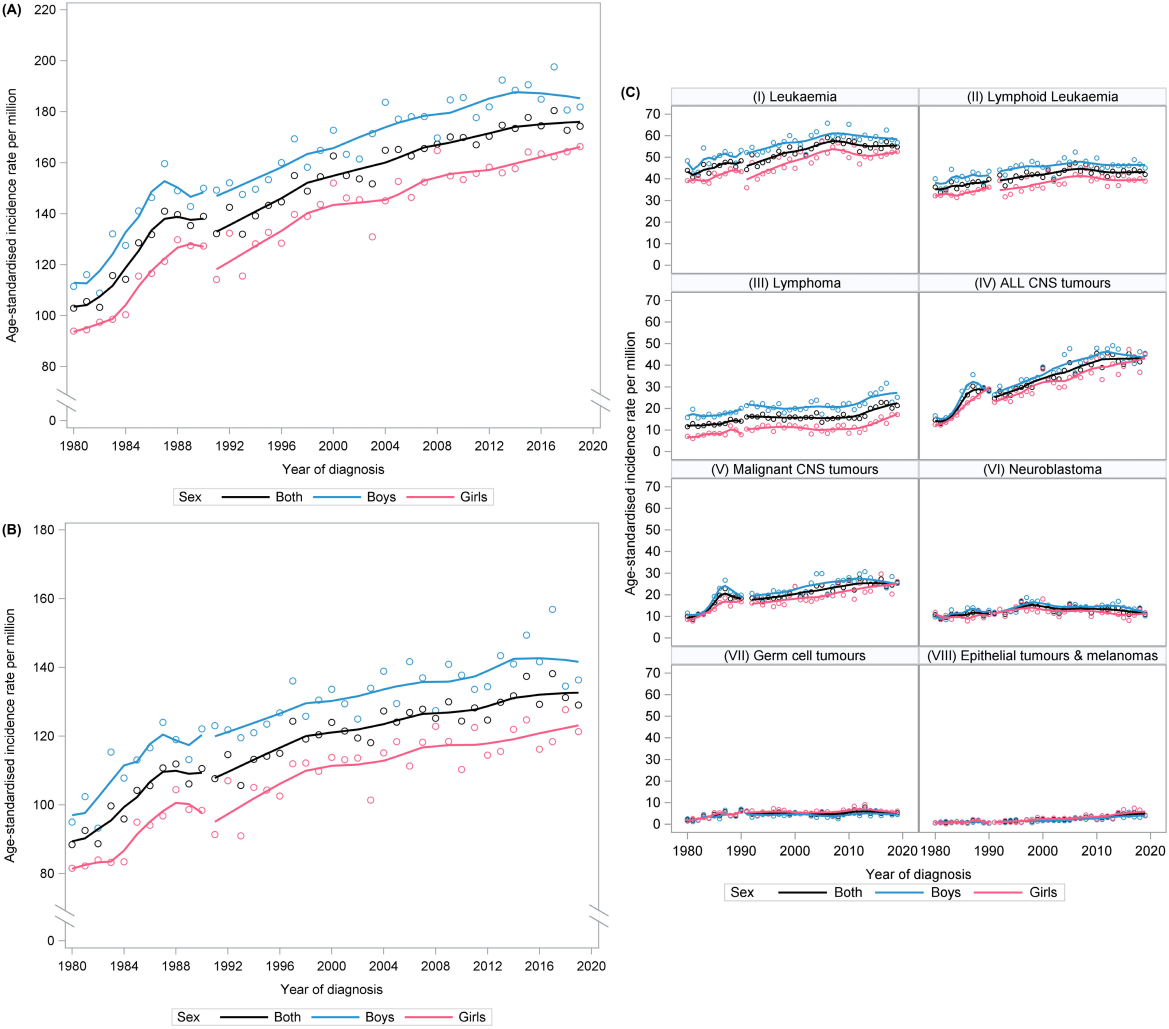
(A) Age‐standardised incidence rates (using Segi World Standard Population) of childhood cancer (diagnosed at ages 0–14 years, all cancer diagnoses combined) in Germany, 1980–2019. (B) Age‐standardised incidence rates (using Segi World Standard Population) of childhood cancer (diagnosed at ages 0–14 years) with tumours of the central nervous system excluded, in Germany, 1980–2019. (C) Age‐standardised incidence rates (using Segi World Standard Population) of (I) leukaemia, (II) lymphoid leukaemia, (III) lymphoma, (IV) CNS tumours, (V) malignant CNS tumours, (VI) neuroblastoma, (VII) germ cell tumours and (VIII) epithelial tumours and melanomas in children aged 0–14 years in Germany, 1980–2019.

**FIGURE 2:**
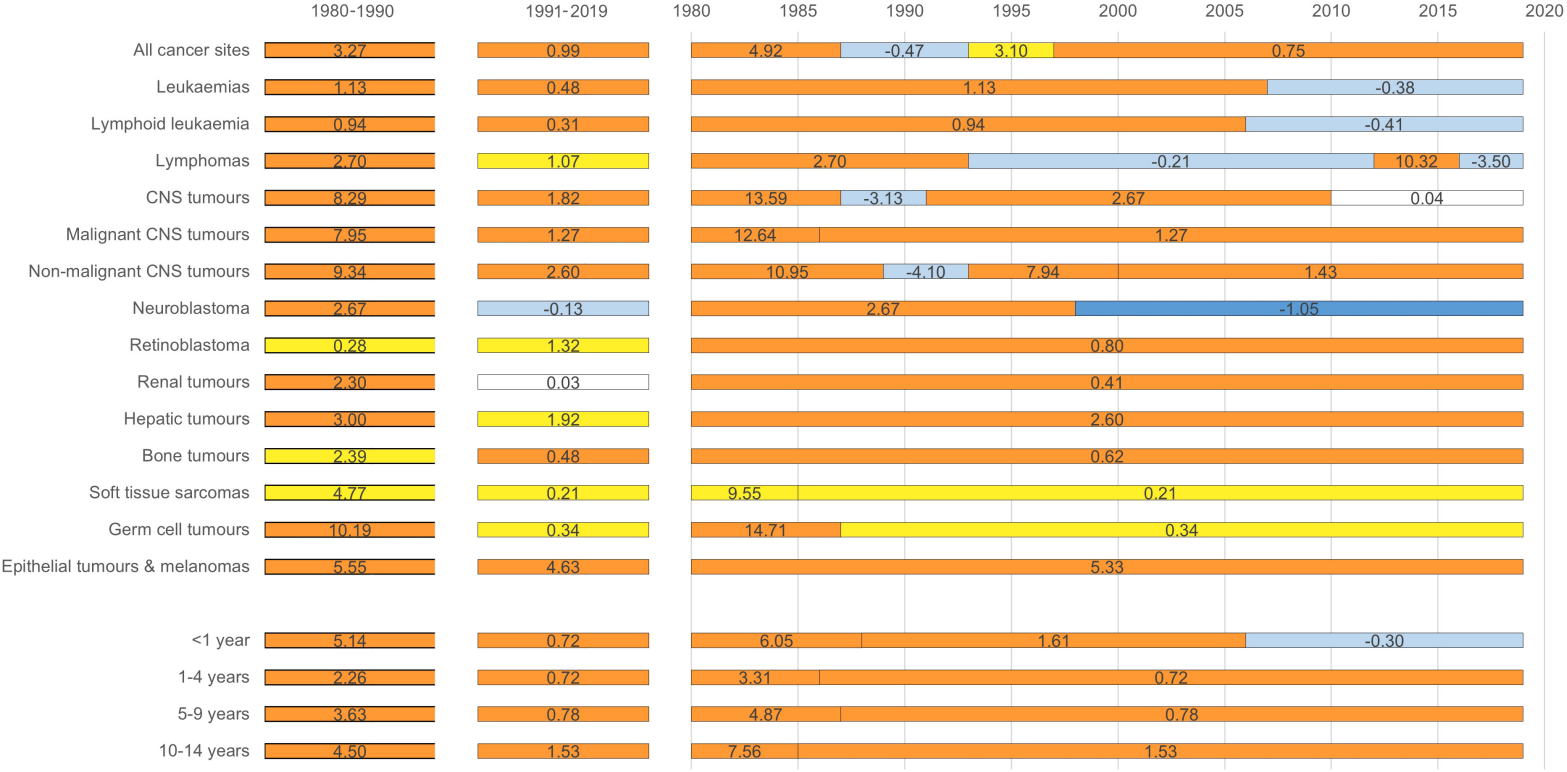
Incidence trends of cancer in children aged 0–14 years in Germany in 1980–1990 (only based on cases from the area of former Western Germany), 1991–2019, and for any time segments identified in Joinpoint regression analysis with respective AAPCs (1980–1990 and 1991–2019) and APCs expressed in %, by diagnostic group and age at diagnosis.

Figure 1A–C visually illustrates the temporal trends in age‐standardised incidence rates (ASRs) for (A) all childhood cancers combined, (B) all childhood cancers excluding CNS tumours and (C) selected diagnostic groups. Figure 2 and Table S1, Supporting Information present the results of the statistical time trend analyses using Joinpoint regression models. The ASRs for childhood cancer overall increased on average by 3.3% annually during 1980–1990 (build‐up phase of the registry) and by 1.0% since 1991 (Figure 2). The trend varied markedly over time, with the steepest increase seen for the first years of registration (APC 1980–1987 = 4.9%; 95% CI: 3.6; 6.3), followed by some years of stable rates, a short period with an APC of 3.1% and a slight, steady increase of 0.8% per year since 1997. Although the formal trend analysis did not identify any joinpoint, the visual presentation of the ASRs over time (Figure 1A) suggests a recent stabilisation in the overall incidence of childhood cancer.

The overall tendency of increasing ASRs as well as the sharp increase during the first years of registration was evident across diagnostic groups and age groups (Figure 2). The steep increase during the 1980s was particularly pronounced for children aged 10–14 years at diagnosis (APC 1980–1985 = 7.6%; 95% CI: 3.8; 11.4) and for children diagnosed as infants (APC 1980–1988 = 6.1%; 95% CI: 3.3; 8.8). By diagnostic group, the steepest increases were seen for malignant CNS tumours (APC 1980–1986 = 12.6%; 95% CI: 5.8; 20.0), non‐malignant CNS tumours (APC 1980–1989 = 11.0%; 95% CI: 6.8; 15.3), soft tissue sarcoma (APC 1980–1985 = 9.6%; 95% CI: −0.5; 20.7) and germ cell tumours (APC 1980–1987 = 14.7%; 95% CI: 4.1; 26.4).

Since the 1990s, temporal patterns were more heterogeneous across diagnostic groups, yet overall, less pronounced than during the 1980s. The APC of ASRs for leukaemia increased steadily until the year 2007 (APC = 1.1%, 95% CI: 0.9; 1.3), where the Joinpoint regression revealed a change in trends towards a slightly decreasing tendency during more recent years (APC = −0.4%, 95% CI: −1.0; 0.2) (Figures 1C and 2 and Table S1). On the contrary, ASRs of most solid tumours continued to rise somewhat over time. The strongest increase, after the late 1980s, was seen for epithelial tumours and melanomas, with an AAPC of 5.3% and no statistically significant break in the temporal pattern over the entire study period. An exception was neuroblastoma, for which incidence rates were rising until the end of the 1990s (APC = 2.7, 95% CI: 1.6; 3.7), followed by a statistically significant decline of −1.1% per year (95% CI: −1.8; −0.3) since 1998 (Figures 1C and 2 and Table S1).

Similar to the early years of registration, the annual increase was somewhat stronger among children diagnosed as infants (APC for 1988–2006 = 1.6%, 95% CI: 1.0; 2.2) and those aged 10–14 years (APC for 1985–2019 = 1.5%, 95% CI: 1.4; 1.7) compared to children diagnosed at ages 1–9 years (Figure 2 and Table S1), but confidence intervals were wide and overlapped.

Time trends were generally similar for boys and girls, with some exceptions due to small numbers of cases for some diagnostic groups, as well as a recent change in the trend for malignant CNS tumours among boys (APC of −2.0% [95% CI: −5.6; 1.8] since 2012) (Table S1).

**FIGURE 3:**
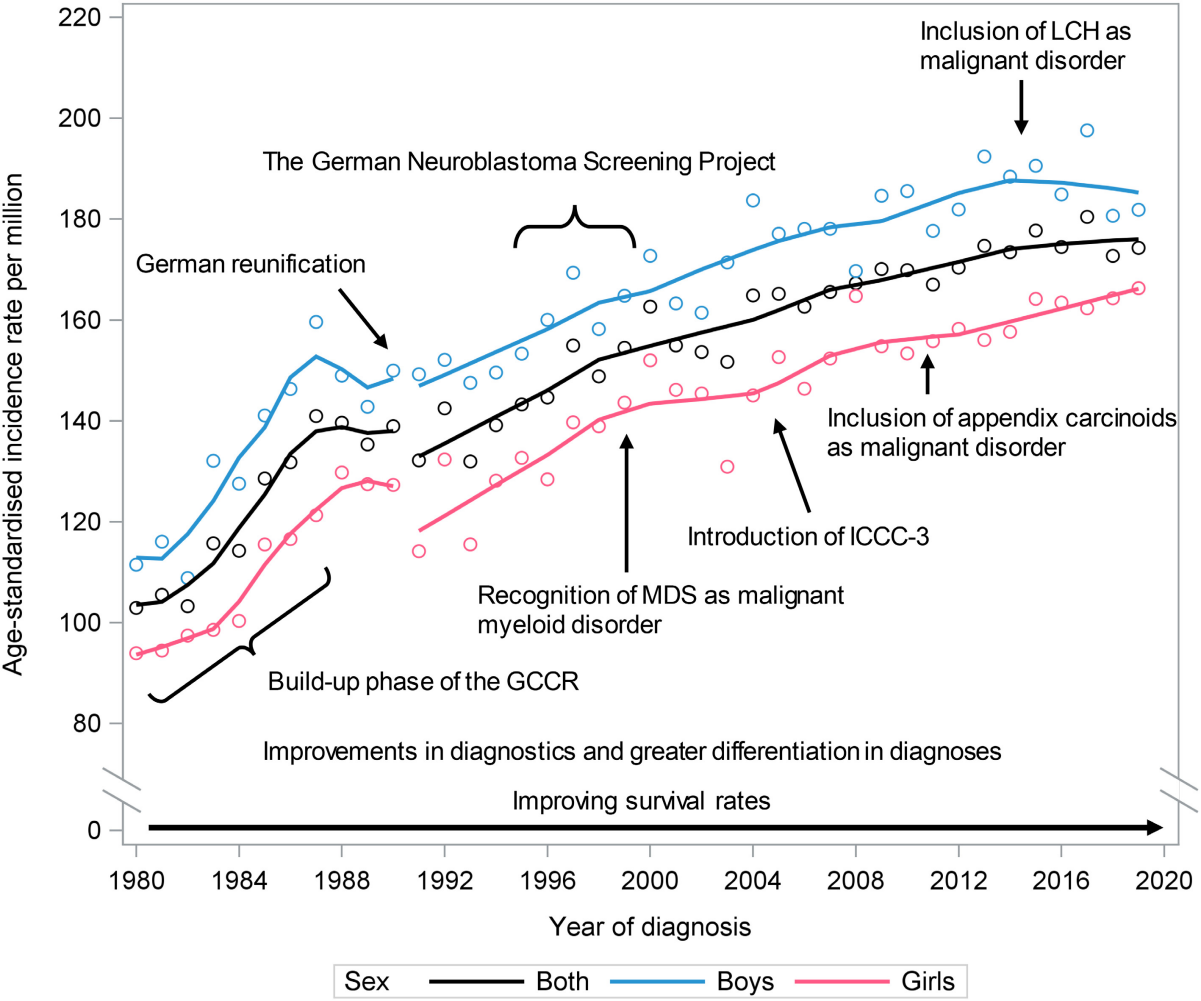
Age‐standardised incidence of childhood cancer in Germany, 1980–2019, and contributing factors over time.

The sensitivity analysis, which excluded cases not previously classified as childhood cancers, revealed overall similar results to those of the main analysis. Only for lymphomas was a minor change in the pattern observed. The previously noted short‐term increase between 2012 and 2016 (see Figure 3)—although based on small numbers, as indicated by the wide confidence intervals—was no longer evident, and no joinpoint was detected after 1992.

## 4 DISCUSSION

With this report, we provide the first long‐term assessment of temporal trends in incidence rates of childhood cancer in Germany, covering a 40‐year period and a study population representing more than 450 million person‐years at risk. As such, this is one of the largest reports worldwide on observed temporal trends, providing valuable insights into reporting patterns and trends across diagnostic subtypes of these rare cancers.

Across the entire period from 1980 to 2019, ASRs were highest for leukaemias, CNS tumours and lymphomas, with boys being somewhat more frequently diagnosed than girls. The age distribution of patients varied markedly between diagnostic groups, with the highest age‐specific incidence rates for all cancers combined observed in infants and children aged 1–4 years. In the most recent diagnostic period (2010–2019), on average 1834 children aged 0–14 years were diagnosed with cancer each year in Germany.

Trend analyses revealed a statistically significant increase in the observed ASRs for childhood cancer overall, from 122.8 per million in 1980–1989 to 173.4 per million in 2010–2019, as well as across age groups and virtually all diagnostic groups. The steepest increase (on average 4.9% per year for all cancers combined) occurred in the initial years of registration (1980–1987), driven primarily by a sharp rise in the ASRs for CNS tumours, soft tissue sarcomas and germ cell tumours. Since the 1990s, temporal patterns were more heterogeneous across diagnostic groups, but have generally been less pronounced compared to the 1980s. The incidence of leukaemia continued to rise slowly until 2007, followed by a slight decline, while the ASRs for solid tumours have continued to increase gradually to the present day. An exception is neuroblastoma, for which the observed incidence rates began to decline gradually starting in 1998.

From an international perspective, the current ASR of 173.4 per million for overall childhood cancer in Germany (in 2010–2019) ranks among the highest in Europe^2^, ^3^, ^4^, ^33^, ^34^, ^35^, ^36^, ^37^, ^38^, ^39^ and is comparable to those observed in Australia^3^ and the United States.^4^ Throughout the 40‐year study period, boys had a higher overall incidence of childhood cancer than girls in Germany, as observed also in other countries.^2^ The three most common diagnostic groups were leukaemias, lymphomas and CNS tumours and the age‐specific incidence rates varied considerably across diagnostic groups, similar to observations in other European and high‐income countries worlwide.^2^, ^3^, ^33^, ^35^, ^37^, ^38^ Of note, international comparisons are notoriously hampered by differences in the diagnostic periods studied, registration procedures, data quality and completeness, as well as random variation arising from the small numbers of cases in specific diagnostic subtypes. For a large number of European countries, North America and Oceania, a gradual increase in childhood cancer incidence rates was reported over the last three decades of the 20th century, with however large variations by region and cancer type.^3^, ^33^, ^35^, ^37^, ^38^, ^40^, ^41^ More recent patterns appear more heterogeneous, with observations of plateaued incidence rates,^37^, ^40^, ^42^, ^43^ while others continue to observe rising trends^3^, ^4^, ^40^ and often marked differences by diagnostic group.^3^, ^4^, ^33^, ^40^ In Germany, we also observed more varied and less pronounced temporal patterns across diagnostic groups since the early 1990s. While the ASRs for solid tumours have continued to increase gradually to the present day (except for neuroblastoma), the incidence rates for leukaemia have shown a slight decreasing tendency. Moreover, although Joinpoint regression did not identify any statistically significant changes in trends, the visual inspection of ASRs suggests also a recent stabilisation in the overall incidence of childhood cancer.

The global increase in incidence rates is considered to be, at least in part, attributable to more complete cancer registration and advances in diagnostic technologies. Historical underascertainment and underreporting in many countries may have improved over time, leading to more complete and accurate data today.^44^ Similarly, the findings of the present analysis from Germany must be interpreted in the context of some cancer registration artefacts and other contributing factors, which do not necessarily reflect a true increase in cancer risk.

First, the steep increase in ASRs during the early years of registration is primarily attributable to improvements in registration completeness, particularly pronounced for CNS tumours and rare tumours, and to a lesser extent for leukaemias, which were as already well reported from the outset (mostly due to the early integration of clinical trials as frontline care for children with leukaemia in the early 1980s). As a result, most diagnostic groups showed a markedly inflated upward trend in ASRs during this period, reflecting artefactual rather than true increases in incidence. However, a minor contribution of true increases cannot be ruled out due to the pronounced effects of the start‐up patterns.

Second, the temporal incidence trends of childhood CNS tumours in Germany have been discussed elsewhere,^45^ with conclusions pointing to a high likelihood of considerable under‐registration due to cases treated in adult oncological or neuro‐oncological facilities, which do not always report reliably to the GCCR. Observations from the time of the COVID‐19 pandemic further suggest that under‐registration likely still occurred in 2019, primarily affecting non‐malignant CNS tumours.^26^ While the GCCR reports completeness levels of over 95% for all other neoplasms combined since the late 1980s, achieving comparable completeness for paediatric CNS tumours remains a challenge.^27^, ^45^

Third, the German reunification may have influenced childhood cancer incidence rates, as it brought together two previously separated populations with starkly different sociocultural circumstances, lifestyles and health care systems, and strong restrictions on exchange. Our analysis of temporal trends showed no marked impact, perhaps because the childhood population of West Germany accounted for more than 80% of all children and thus largely shaped the combined trends. CNS tumours represent a notable exception, hampered by reporting artefacts as described above, for which Joinpoint regression identified a significant breakpoint in 1991. In addition, previously published observations revealed lower ASRs for lymphoblastic leukaemia in the former German Democratic Republic, which rose rapidly following reunification.^46^

Fourth, for neuroblastoma specifically, the German Neuroblastoma Screening Project for infants, conducted in Germany from May 1995 to April 2001,^47^ had a significant influence on the observed incidence patterns in Germany during the late 1990s and early 2000s. The screening programme was initiated to assess whether screening asymptomatic infants for neuroblastoma would lead to a decrease in advanced‐stage neuroblastoma diagnoses (which have a significantly worse prognosis than early‐stage disease) and improve survival probabilities. It was carried out in six federal states, while the remaining parts of Germany served as the control group. The conclusion from the German screening project was that although early detection of neuroblastomas increased, there was no significant improvement in the incidence of metastatic disease nor overall survival probabilities. Moreover, a substantial number of detected cases were of tumours that might have regressed spontaneously without intervention,^47^ indicating considerable overdiagnosis. The overdiagnosis of cases likely explains most of the rising neuroblastoma incidence observed until the end of the 1990s (2.7% per year, 95% CI: 1.6; 3.7), as well as the decreasing trend afterward (−1.1% per year, 95% CI: −1.8; −0.3), owing to both the typical shift in timepoint of diagnosis among screened children, as well as the discontinuation of the program.

Fifth, over the 40‐year period, the classification system for childhood cancers has been updated multiple times. These updates have considered improvements in diagnostics and greater differentiation in diagnoses and led to the inclusion of certain types of neoplasia previously not eligible for registration; however, their numbers were small and had rather little impact on the overall incidence rates, but have somewhat impacted the temporal patterns of certain specific cancer subtypes. Most importantly, MDS was not traditionally classified as a childhood cancer in epidemiological studies or cancer registries until the early 2000s. With the introduction of ICD‐O‐3 (replacing ICD‐O‐2), MDS was recognized as a malignant myeloid disorder and subsequently included in ICCC‐3 under Group I: Leukaemias, specifically as subtype I(d): Myelodysplastic syndrome and other myeloproliferative diseases.^28^ Similarly, with the introduction of ICD‐O‐3.1, the first revision of ICD‐O‐3, all types of LCH have been recognised as malignant and, since 2014, are included under subgroup II(d): Miscellaneous lymphoreticular neoplasms.^28^ Finally, in line with the introduction of ICD‐O‐3.1, appendix carcinoids were reclassified as malignant in 2011 and are now included in Group XI: Other malignant epithelial neoplasms and malignant melanomas, specifically under subgroup XI(f): Other and unspecified carcinomas.^28^ However, the sensitivity analysis yielded overall results that were similar to those of the main analysis, with the exception for lymphomas. The reclassification of LCH likely contributed to minor changes in incidence rates, which in turn led to slight changes in the results of the time trend analysis, although these were based on small numbers. Further, the one‐to‐one reclassification of diagnoses based on earlier ICCC versions to ICCC‐3 involved some specific diagnoses that were difficult to unequivocally reclassify.

Finally, another minor contributing factor may be the remarkable advances in survival rates over the past five decades, with current survival rates exceeding 85%.^48^ The continuous improvements in survival have led to a longer time at risk and, consequently, a slight increase in the number of SPNs diagnosed in childhood (and thus registered at GCCR and part of this analysis), predominantly related to the prior cancer treatments (e.g., subsequent MDS/t‐AML, CNS tumours, sarcoma) and a myriad of rare genetic syndromes that confer predisposition to specific combinations of (childhood) cancers.^23^

Regrettably, the effects of changes in exposure to aetiological factors—and thus changes in actual risk—cannot be quantified with this descriptive assessment. In particular, the underlying causes of the increasingly heterogeneous temporal patterns observed in recent years remain unexplained but may, to some extent, reflect changes in environmental exposures that could have contributed to genuine shifts in the risk of specific childhood cancers.^6^, ^7^, ^8^, ^9^, ^10^, ^11^, ^12^, ^13^, ^14^, ^17^ The early age at diagnosis suggests an inherited component, and several critical exposure time windows have been proposed, including parental exposures prior to conception, in utero exposures during pregnancy and exposures during infancy and early childhood; all of which may play a role in the development of childhood cancers.^10^, ^14^

### 4.1 Strengths and limitations

Significant strengths of our study include the exceptionally long observation period of 40 years, the high‐quality data from the GCCR, which has been operating since 1980s, and the use of Germany as the study setting. The GCCR is one of the longest‐standing nationwide population‐based childhood cancer registries worldwide. Germany provides universal healthcare access regardless of socioeconomic background and benefits from a sizable child population of approximately 11.5 million (as of the end of 2019). This results in a substantial number of cases, allowing meaningful analyses despite the comparatively rare occurrence of specific childhood cancer types and offers an up‐to‐date picture of diagnosis‐specific distribution and temporal patterns.

A limitation of the study is the incomplete information on disease stage and grading.^49^ Analyses of time trends by stage and grading could have provided additional insights,^49^ such as whether the observed increase in ASRs for malignant CNS tumours or epithelial neoplasms was specific to or most pronounced in early‐stage diagnoses.^50^

## 5 CONCLUSIONS

Over the past 40 years, childhood cancer incidence rates have shown increasing trends in Germany. After a steep rise during the early years of registration (1980–1987), primarily attributable to improvements in registration completeness, temporal patterns were more heterogeneous across diagnostic groups; yet overall, less pronounced than during the 1980s.

This comprehensive assessment of childhood cancer patterns and temporal developments in Germany provides insight into the daily challenges of as precise as possible registration for rare cancers. Building and maintaining virtually complete reporting and registration, marked changes in the population under observation, improvements in diagnostics and greater diagnostic differentiation, as well as changes in cancer classification systems, all influence the observed incidence rates. However, thanks to the large dataset, these effects were to some extent quantifiable. This calls for caution and humility when interpreting temporal patterns coming from smaller target populations and over shorter time periods, before too quickly attributing changes in patterns to changes in underlying risk and related risk factors. Many observations remained relatively stable over time in our data, such as the ranking of diagnostic subtypes, the dominance of haematological malignancies, age distributions, and sex ratios, and notably also the incidence rate over the past 12 years of the most common subtype, leukaemia. Some temporal patterns remain unexplained, such as the ups and downs in the incidence of lymphoma and the gradual increase in some non‐CNS rare solid cancers. Continuous monitoring is essential to project needed capacities and resources for diagnosis, treatment and after‐care, as well as early alerts if any changes in patterns occur to identify their causes as quickly as possible for action.

## DISCLAIMER

Where authors are identified as personnel of the International Agency for Research on Cancer/World Health Organization, the authors alone are responsible for the views expressed in this article and they do not necessarily represent the decisions, policy or views of the International Agency for Research on Cancer/World Health Organization.

## AUTHOR CONTRIBUTIONS

**Friederike Erdmann:** Conceptualization; writing – original draft; data curation; methodology; investigation; visualization; project administration. **Maike Wellbrock:** Methodology; formal analysis; investigation; visualization; writing – review and editing. **Claudia Trübenbach:** Data curation; formal analysis; writing – review and editing. **Desiree Grabow:** Data curation; writing – review and editing. **Martin Schrappe:** Writing – review and editing; data curation. **Peter Kaatsch:** Writing – review and editing; data curation. **Claudia Spix:** Data curation; methodology; writing – review and editing; investigation. **Joachim Schüz:** Conceptualization; investigation; methodology; writing – review and editing. **Cécile M. Ronckers:** Data curation; investigation; methodology; writing – review and editing.

## CONFLICT OF INTEREST STATEMENT

The authors declare no conflicts of interest.

## ETHICS STATEMENT

No ethics approval or consent was required for this study, as no active participation of patients was required. This research was carried out in compliance with the requirements of the General Data Protection Regulation (GDPR) and in accordance with The Code of Ethics of the World Medical Association (Declaration of Helsinki) for experiments involving humans.

## Supporting information


**Table S1.** Annual percentage changes (APC) in the age‐standardised incidence of childhood cancers in Germany in 1980–2019, by diagnostic group, age at diagnosis and sex.

## Data Availability

The data that support the findings of this study may be available on aggregated or pseudonymised individual‐level from the corresponding author upon reasonable request, in compliance with the national data protection requirements.
