# Supplementary material for: Incidence patterns and temporal trends of childhood cancer in Germany, 1980–2019: Forty years of childhood cancer registration in Germany
Source: Int J Cancer. 2025 Sep 29;158(6):1541–54. doi: 10.1002/ijc.70167 (PMC12811210; doi:10.1002/ijc.70167)
Supplement: Supplementary file 1 — Table S1. Annual percentage changes (APC) in the age‐standardised incidence of childhood cancers in Germany in 1980–2019, by diagnostic group, age at diagnosis and sex. [file IJC-158-1541-s001.pdf]

Incidence patterns and temporal trends of childhood cancer in Germany, 1980 – 2019: 40 years of childhood cancer registration in Germany

Friederike Erdmann,Maike Wellbrock, Claudia Trübenbach, Desiree Grabow, Martin Schrappe, Peter Kaatsch, Claudia Spix, Joachim Schüz, Cécile M. Ronckers

Content **Pages**

Table S1:
Annual percentage changes (APC) in the age-standardised incidence of childhood cancers in Germany in 1980-2019, by diagnostic group, age at diagnosis and sex. 1-3

Table S1: Annual percentage changes (APC)[a] in the age-standardised incidence of childhood cancers in Germany in 1980-2019, by diagnostic group, age at diagnosis and sex.

	Diagnostic group[b]	1 Period	1 APC (%) (95% CI)[c,]	2 Period	2 APC (%) (95% CI)[c]	3 Period	3 APC (%) (95% CI)[c]	4 Period	4 APC (%) (95% CI)[c]
ALL CHILDREN	All cancer diagnoses	1980-1987	4.92 (3.6; 6.3)	1987-1993	-0.47 (-2.3; 1.4)	1993-1997	3.10 (-0.7; 7.0)	1997-2019	0.75 (0.6; 0.9)
	Leukaemias	1980-2007	1.13 (0.9; 1.3)	2007-2019	-0.38 (-1.0; 0.2)				
	Lymphoid leukaemia	1980-2006	0.94 (0.7; 1.2)	2006-2019	-0.41 (-1.0; 0.2)				
	Lymphoma	1980-1993	2.70 (1.6; 3.8)	1993-2012	-0.21 (-0.8; 0.4)	2012-2016	10.32 (0.7; 20.9)	2016-2019	-3.50 (-11.2; 4.8)
	CNS tumours	1980-1987	13.59 (9.3; 18.0)	1987-1991	-3.13 (-13.8; 8.9)	1991-2010	2.67 (2.0; 3.3)	2010-2019	0.04 (-1.6; 1.6)
	Malignant	1980-1986	12.64 (5.8; 20.0)	1986-2019	1.27 (0.9; 1.6)				
	Non-malignant	1980-1989	10.95 (6.8; 15.3)	1989-1993	-4.10 (-17.6; 11.6)	1993-2000	7.94 (3.2; 12.8)	2000-2019	1.43 (0.7; 2.1)
	Neuroblastoma	1980-1998	2.67 (1.6; 3.7)	1998-2019	-1.05 (-1.8; -0.3)				
	Retinoblastoma	1980-2019	0.80 (0.2; 1.4)						
	Renal tumours	1980-2019	0.41 (0.1; 0.7)						
	Hepatic tumours	1980-2019	2.60 (1.8; 3.4)						
	Bone tumours	1980-2019	0.62 (0.3; 1.0)						
	Soft tissue sarcomas	1980-1985	9.55 (-0.5; 20.7)	1985-2019	0.21 (-0.2; 0.6)				
	Germ cell tumours	1980-1987	14.71 (4.1; 26.4)	1987-2019	0.34 (-0.3; 1.0)				
	Epithelial tumours & melanomas	1980-2019	5.33 (4.6; 6.0)						
	Other malignant neoplasms	n.a.							
	<1 year	1980-1988	6.05 (3.3; 8.8)	1988-2006	1.61 (1.0; 2.2)	2006-2019	-0.30 (-1.2; 0.6)		
	1-4 years	1980-1986	3.31 (0.2; 6.5)	1986-2019	0.72 (0.5; 0.9)				
	5-9 years	1980-1987	4.87 (2.5; 7.3)	1987-2019	0.78 (0.6; 1.0)				
	10-14 years	1980-1985	7.56 (3.8; 11.4)	1985-2019	1.53 (1.4; 1.7)				
BOYS	All cancer diagnoses	1980-1986	5.10 (2.6; 7.7)	1986-2019	0.87 (0.7; 1.0)				
	Leukaemias	1980-2006	1.06 (0.8; 1.3)	2006-2019	-0.34 (-1.0; 0.4)				
	Lymphoid leukaemia	1980-2005	0.87 (0.6; 1.2)	2005-2019	-0.37 (-1.1; 0.3)				
	Lymphoma	1980-1992	2.22 (0.7; 3.8)	1992-2011	-0.01 (-0.7; 0.7)	2011-2017	6.85 (1.7; 12.3)	2017-2019	-12.54 (-29.9; 9.2)
	CNS tumours	1980-1987	14.46 (9.4; 19.8)	1987-1990	-7.10 (-30.4; 24.1)	1990-2010	2.78 (2.1; 3.5)	2010-2019	-0.72 (-2.6; 1.2)

	Malignant	1980-1987	16.00 (9.8; 22.5)	1987-1990	-9.46 (-36.4; 28.8)	1990-2012	2.01 (1.2; 2.8)	2012-2019	-2.01 (-5.6; 1.8)
	Non-malignant	1980-2005	4.63 (3.9; 5.4)	2005-2019	0.90 (-0.2; 2.0)				
	Neuroblastoma	1980-1994	1.28 (-0.5; 3.1)	1994-1997	9.69 (-19.3; 49.0)	1997-2019	-1.13 (-1.9; -0.4)		
	Retinoblastoma	1980-2019	1.03 (0.4; 1.7)						
	Renal tumours	1980-2019	0.24 (-0.2; 0.7)						
	Hepatic tumours	1980-2019	2.28 (1.4; 3.2)						
	Bone tumours	1980-2019	0.73 (0.2; 1.3)						
	Soft tissue sarcomas	1980-2019	0.52 (0.1; 1.0)						
	Germ cell tumours	1980-2019	0.64 (-0.2; 1.5)						
	Epithelial tumours & melanomas	1980-2019	4.60 (3.7; 5.5)						
	Other malignant neoplasms	n.a.							
	<1 year	1980-2003	2.83 (2.0; 3.7)	2003-2019	-0.84 (-2.0; 0.3)				
	1-4 years	1980-2019	0.79 (0.6; 1.0)						
	5-9 years	1980-1987	4.51 (1.8; 7.3)	1987-2019	0.78 (0.6; 1.0)				
	10-14 years	1980-1984	8.72 (2.7; 15.1)	1984-2019	1.38 (1.2; 1.6)				
GIRLS	All cancer diagnoses	1980-1988	3.92 (2.1; 5.8)	1988-2019	1.01 (0.8; 1.2)				
	Leukaemias	1980-2007	1.27 (0.9; 1.6)	2007-2019	-0.32 (-1.3; 0.7)				
	Lymphoid leukaemia	1980-2019	0.65 (0.4; 0.9)						
	Lymphoma	1980-1994	3.86 (1.9; 5.9)	1994-2012	-0.69 (-1.9; 0.5)	2012-2019	8.53 (3.9; 13.5)		
	CNS tumours	1980-1987	11.14 (4.6; 18.1)	1987-2019	1.95 (1.6; 2.4)				
	Malignant	1980-1986	11.10 (1.7; 21.4)	1986-2019	1.57 (1.1; 2.0)				
	Non-malignant	1980-1990	13.00 (8.3; 17.9)	1990-1993	-16.24 (-44.26; 26.5)	1993-1996	23.06 (-16.1; 80.5)	1996-2019	1.74 (1.0; 2.5)
	Neuroblastoma	1980-1996	2.87 (1.1; 4.6)	1996-2019	-1.05 (-2.9; -0.1)				
	Retinoblastoma	1980-2019	0.48 (-0.3; 1.3)						
	Renal tumours	1980-2016	0.83 (0.4; 1.3)	2016-2019	-11.53 (-25.8; 5.5)				
	Hepatic tumours	1980-2019	2.58 (1.3; 3.9)						
	Bone tumours	1980-2019	0.49 (0.0; 1.0)						
	Soft tissue sarcomas	1980-2019	0.57 (0.0; 1.1)						

Germ cell tumours	1980- 1986	21.16 (6.0; 38.5)	1986-2019	0.64 (-0.0; 1.3)				
Epithelial tumours & melanomas	1980-2019	5.76 (4.7; 6.9)						
Other malignant neoplasms	n.a							
<1 year	1980-1988	6.83 (2.7; 11.1)	1988-2019	1.01 (0.7; 1.4)				
1-4 years	1980-2019	0.93 (0.7; 1.2)						
5-9 years	1980-1998	4.67 (1.3; 8.1)	1988-2019	0.74 (0.4; 1.1)				
10-14 years	1980-1988	7.80 (5.2; 10.4)	1988-1991	-7.01 (-26.2; 17.2)	1991-1999	3.95 (1.5; 6.5)	1999-2019	1.39 (0.9; 1.8)

[a]Calculated with Joinpoint Regression Software, maximum of 4 joinpoints allowed.
[b]Diagnostic groups defined according to the International Classification of Childhood Cancer – 3rd edition (ICCC-3).
[c]Based on age-standardised incidence rate (standardized according to the Segi World Standard Population) per 1,000,000 population aged 0-14 years.
n.a.: not applicable – the APCs could not be calculated due to zero counts in at least one cell.
